# Response to: gender differences on neuromuscular strategy during drop jump: a comment on Helm et al. (2019) by Di Giminiani et al.

**DOI:** 10.1007/s00421-020-04460-z

**Published:** 2020-08-09

**Authors:** M. Helm, K. Freyler, J. Waldvogel, A. Gollhofer, R. Ritzmann

**Affiliations:** 1 Institute of Sport and Sport Science, University of Freiburg, Schwarzwaldstraße 175, 79117 Freiburg, Germany; 2 Rennbahnklinik, Muttenz, Switzerland

Dear Editor,

We are very grateful to receive the comment from Di Giminiani et al. (2020). Based on the previously described gender differences, they suggest that we should clarify if sex specificity exists in kinetic, kinematic and motor control strategies in the stretch–shortening cycle (SSC) in our study. Although sex specificity was not the major hypothesis-driven interest of our study, we found the recommendation relevant for our research. Therefore, we decided to recalculate the statistics according to the author’s suggestions to verify or neglect gender differences. Thereby, we have included gender as a between-subject factor to the two-factor repeated measures analysis of variance (rmANOVA) [anticipation (2) × drop height (3)], because gender does not match the requirements of an ANCOVA (covariate: metric scale level). This extended statistical analyses show no statistically significant effect of gender, neither the interaction gender * drop height nor the interaction gender * anticipation regarding the performance parameters, the kinematics, the kinetics and the EMG activity in seven of the eight measured muscles. The only exception is the EMG activity of the VM during the SLR phase (see Supplementary Tables 1–3).

Hence, all the other muscles and phases as well as the mechanical parameters are unaffected by gender during the SSC. The SSC is characterized by a stretching of a pre-activated muscle–tendon unit, immediately followed by a contraction of the muscle (Komi 1984). Regarding the role of different leg muscles during the SSC, the VM muscle serves as a knee extensor. However, it has been documented that the most relevant muscles within the SSC are in particular the triceps surae muscles serving as plantarflexors, which, together with the Achilles tendon, are the primary players allowing to efficiently store and release elastic energy (Komi and Gollhofer 1997; Arampatzis et al. 2001). Based on this, and including that the task instruction was to keep a stiff knee during ground contact, we conclude that in our study, the VM muscle plays a minor role during SSC and consequently the neuromuscular differences between gender in VM EMG activity during SLR are functionally less relevant. This also becomes apparent as the kinematic and performance parameters do not show any gender differences. We assume that possibly load- and task-specific differences in our study as well as differences in the subject population compared to studies cited by Di Giminiani et al. (2020) could have contributed to the absence of gender-specific findings.

The absence of significances clearly demonstrates that gender has a marginal effect on the interpretation of our results and hence, that within our investigated subject population, this effect is negligible for the outcome of our study. Future research analyzing greater subject populations might provide the research community with better understandings into sex-specific variations during the SSC.

## Electronic supplementary material

Below is the link to the electronic supplementary material.

Supplementary file1 (DOCX 40 kb)
