# Supplementary material for: Response to: gender differences on neuromuscular strategy during drop jump: a comment on Helm et al. (2019) by Di Giminiani et al
Source: Eur J Appl Physiol. 2020 Aug 9;120(11):2557–8. doi: 10.1007/s00421-020-04460-z (PMC7560930; doi:10.1007/s00421-020-04460-z)
Supplement: Supplementary file 1 — Supplementary file1 (DOCX 40 kb) [file 421_2020_4460_MOESM1_ESM.docx]

**Supplementary material**

Table 1: Effect of gender on performance parameters during the stretch-shortening cycle: F_max_/BW, leg stiffness, jump height as well as maximal COM displacement.

| **Parameter** | **Gender** | ***Low*** | | | | ***Medium*** | | | ***High*** | | | *Statistics – rmANOVA (P, F,*$\eta^{2}$_p_*)* | |
| --- | --- | --- | --- | --- | --- | --- | --- | --- | --- | --- | --- | --- | --- |
| **Force** |  | | Known | Unknown | Cheat | | Known | Unknown | | Known | Unknown | *Interaction effect: anticipation*gender* | *Interaction effect: drop height*gender* |
| **F_max_/BW (N/kg)** | Male | | 23.0 ± 4.2 | 23.3 ± 3.9 | 28.0 ± 9.0 | | 26.5 ± 5.3 | 22.6 ± 2.4 | | 32.7 ± 7.4 | 32.7 ± 4.5 | F(1, 16)=0.08, p=0.78, $\eta^{2}$_p_=0.01 | F(1.44, 23.05)=0.87, p=0.4, $\eta^{2}$_p_=0.05 |
|  | Female | | 25.2 ± 6.1 | 26.8 ± 7.5 | 30.1 ± 16.2 | | 31.0 ± 7.8 | 26.5 ± 6.9 | | 38.1 ± 11.6 | 38.5 ± 12.9 |  |  |
| **Performance parameters** |  | | Known | Unknown | Cheat | | Known | Unknown | | Known | Unknown | *Interaction effect: anticipation*gender* | *Interaction effect: drop height*gender* |
| **Leg stiffness (N/m)** | Male | | 13040.3 ± 4010.4 | 10692.9 ± 3637.6 | 9150.2 ± 2993.5 | | 15010.6 ± 5401.6 | 11459.1 ± 4095.6 | | 18046.0 ± 5833.0 | 16693.0 ± 5807.4 | F(1, 16) =0.54, p=0.47, $\eta^{2}$_p_=0.03 | F(1.19, 19.04) =1.66, p=0.22, $\eta^{2}$_p_=0.09 |
|  | Female | | 12209.7 ± 4017.8 | 9050.9 ± 2392.9 | 7350.3 ± 2094.8 | | 16285.2 ± 6216.9 | 11045.7 ± 4612.7 | | 19590.1 ± 7906.3 | 18147.9 ± 7894.5 |  |  |
| **Jump height (cm)** | Male | | 12.4 ± 5.4 | 9.4 ± 4.3 | 18.2 ± 7.5 | | 15.3 ± 4.2 | 13.3 ± 5.7 | | 14.2 ± 3.2 | 10.5 ± 4.1 | F(1, 20)=0.17, *p*=0.68, η^2^_p_ =0.01 | F(2, 40)=2.11,  p=0.14, η^2^_p_ =0.1 |
|  | Female | | 12.1 ± 4.2 | 9.0 ± 4.9 | 14.5 ± 6.8 | | 12.7 ± 4.9 | 11.0 ± 6.0 | | 11.6 ± 4.6 | 8.9 ± 4.6 |  |  |
| **COM displacement (m)** | Male | | 14.1 ± 3.2 | 16.0 ± 3.9 | 17.1 ± 4.0 | | 14.2 ± 3.0 | 16.5 ± 3.8 | | 14.9 ± 3.1 | 14.8 ± 2.7 | F(1, 16) = 0.24, p=0.63, $\eta^{2}$_p_=0.02 | F(1.12, 17.86) =0.18, p=0.71, $\eta^{2}$_p_=0.01 |
|  | Female | | 12.9 ± 2.1 | 14.8 ± 2.7 | 17.2 ± 3.5 | | 13.1 ± 2.7 | 15.6 ± 3.0 | | 13.8 ± 3.3 | 14.3 ± 3.4 |  |  |

Values are means ± SD. The interaction effects between anticipation * gender and drop height * gender are displayed with corresponding *F* values and *p* values (**p* < 0.05), as well as effect sizes according to $\eta^{2}$_p_ partial eta-squared.

Table 2: The effect of gender on kinematic variables at initial and during ground contact (GC).

| **Parameter** | **Gender** | | ***Low*** | | | | ***Medium*** | | ***High*** | | | *Statistics – rmANOVA (P, F,*$\eta^{2}$_p_*)* | |
| --- | --- | --- | --- | --- | --- | --- | --- | --- | --- | --- | --- | --- | --- |
| **Joint angles**  at initial GC (°) |  | Known | | Unknown | Cheat | Known | | Unknown | | Known | Unknown | *Interaction effect: anticipation*gender* | *Interaction effect: drop height*gender* |
| **Hip joint** | Male | 157.1 ± 11.2 | | 159.5 ± 9.9 | 157.2 ± 9.3 | 157.2 ± 10.4 | | 158.7 ± 10.3 | | 156.7 ± 10.6 | 157.9 ± 9.7 | F(1, 16)=0.94, p=0.35, $\eta^{2}$_p_=0.06 | F(1.3, 22.72)=0.67, p=0.46, $\eta^{2}$_p_=0.04 |
|  | Female | 157.2 ± 5.4 | | 158.6 ± 6.2 | 157.7 ± 8.0 | 156.2 ± 5.2 | | 156.8 ± 5.7 | | 155.3 ± 6.6 | 156.7 ± 6.0 |  |  |
| **Knee joint** | Male | 161.9 ± 7.0 | | 168.1 ± 3.6 | 167.9 ± 5.6 | 156.8 ± 4.7 | | 161.4 ± 4.3 | | 153.8 ± 4.3 | 155.0 ± 4.8 | F(1, 16)=2.28, p=0.15, $\eta^{2}$_p_=0.13 | F(1.16, 18.52)=0.92, p=0.37, $\eta^{2}$_p_=0.05 |
|  | Female | 163.4 ± 5.9 | | 167. 7± 5.3 | 167.6 ± 8.4 | 158.5 ± 3.1 | | 161.6 ± 4.5 | | 156.0 ± 3.0 | 157.1 ± 3.1 |  |  |
| **Ankle joint** | Male | 136.1 ± 5.4 | | 139.1 ± 4.5 | 139.3 ± 3.7 | 135.9 ± 5.4 | | 137.4 ± 4.9 | | 135.9 ± 5.4 | 136.5 ± 5.0 | F(1, 16)=2.48, p=0.14, $\eta^{2}$_p_=0.13 | F(1.12, 17.95)=1.84, p=0.19, $\eta^{2}$_p_=0.1 |
|  | Female | 134.5 ± 5.8 | | 135.9 ± 4.8 | 136.1 ± 9.8 | 135.0 ± 3.6 | | 135.6 ± 3.4 | | 135.8 ± 3.7 | 136.4 ± 3.2 |  |  |
| **Maximal angular excursions** during GC (Δ°) |  | Known | | Unknown | Cheat | Known | | Unknown | | Known | Unknown | *Interaction effect: anticipation*gender* | *Interaction effect: drop height*gender* |
| **Hip angle** | Male | 20.7 ± 6.9 | | 22.0 ± 7.5 | 24.8 ± 8.2 | 19.2 ± 6.5 | | 20.9 ± 7.2 | | 19.6 ± 6.1 | 18.1 ± 6.2 | F(1, 16) =3.5, p=0.08, η²=0.18 | F(1.17, 18.64) =1.26, p=0.28, η²=0.07 |
|  | Female | 22.5 ± 5.9 | | 24.6 ± 5.2 | 30.8 ± 7.9 | 22.1 ± 6.2 | | 25.1 ± 5.7 | | 22.8 ± 6.3 | 22.8 ± 5.6 |  |  |
| **Knee angle** | Male | 47.4 ± 6.6 | | 50.6 ± 7.5 | 57.6 ± 6.2 | 46.1 ± 6.1 | | 50.8 ± 7.5 | | 47.0 ± 9.5 | 45.3 ± 6.5 | F(1, 16) =3.32, p=0.09, η²=0.17 | F(1.28, 20.51)=0.02, p=0.94, η²=0.001 |
|  | Female | 45.5 ± 4.5 | | 50.6 ± 4.3 | 57.6 ± 6.2 | 43.7 ± 6.2 | | 50.8 ± 4.9 | | 44.6 ± 6.9 | 45.3 ± 6.5 |  |  |
| **Ankle angle** | Male | 48.7 ± 6.3 | | 47.8 ± 6.7 | 48.0 ± 9.8 | 49.5 ± 5.5 | | 48.7 ± 6.3 | | 51.4 ± 7.2 | 49.9 ± 5.5 | F(1, 16) =1.11, p=0.31, $\eta^{2}$_p_=0.07 | F(1.66, 26.51) =0.66, p=0.81, $\eta^{2}$_p_=0.01 |
|  | Female | 48.3 ± 7.6 | | 48.4 ± 7.5 | 48.0 ± 17.4 | 50.1 ± 6.0 | | 49.2 ± 6.9 | | 50.8 ± 6.9 | 50.2 ± 6.5 |  |  |
| **Peak angular velocity** during eccentric phase (°/sec) |  | Known | | Unknown | Cheat | Known | | Unknown | | Known | Unknown | *Interaction effect: anticipation*gender* | *Interaction effect: drop height*gender* |
| **Hip flexion** | Male | 17.2 ± 9.0 | | 23.6 ± 7.0 | 36.8 ± 18.8 | 17.3 ± 6.9 | | 21.3 ± 7.0 | | 20.9 ± 8.6 | 21.0 ± 8.2 | F(1, 16) =0.003, p=0.96, $\eta^{2}$_p_=<0.001 | F(.23, 19.7) =1.33, p=0.27, $\eta^{2}$_p_=0.08 |
|  | Female | 21.6 ± 4.6 | | 28.3 ± 4.8 | 31.0 ± 5.9 | 23.3 ± 4.9 | | 26.6 ± 4.6 | | 29.4 ± 6.6 | 30.0 ± 6.1 |  |  |
| **Knee flexion** | Male | 46.3 ± 7.9 | | 54.8 ± 8.8 | 65.7 ± 13.7 | 41.6 ± 5.6 | | 50.0 ± 5.5 | | 42.7 ± 7.9 | 42.9 ± 7.4 | F(1, 16) =0.96, p=0.34, $\eta^{2}$_p_=0.06 | F(1.32, 21.06)=0.94, p=0.37, $\eta^{2}$_p_=0.06 |
|  | Female | 51.4 ± 6.8 | | 62.5 ± 8.5 | 66.5 ± 15.7 | 44.4 ± 6.9 | | 54.0 ± 7.3 | | 45.4 ± 10.3 | 47.3 ± 8.7 |  |  |
| **Ankle plantarflexion** | Male | 70.9 ± 5.2 | | 74.0 ± 4.9 | 73.3 ± 5.3 | 77.2 ± 5.8 | | 79.7 ± 5.3 | | 88.1 ± 7.0 | 90.4 ± 5.7 | F(1, 16) =1.88, p=0.19, $\eta^{2}$_p_=0.11 | F(1.47, 23.53) =3.09, p=0.06, $\eta^{2}$_p_=0.16 |
|  | Female | 73.6 ± 4.7 | | 77.8 ± 3.1 | 76.5 ± 22.1 | 8.4 ± 5.4 | | 83.0 ± 5.7 | | 9.5 ± 7.0 | 94.8 ± 7.1 |  |  |

Values are means ± SD. The interaction effects between anticipation * gender and drop height * gender are displayed with corresponding *F* values and *p* values (**p* < 0.05), as well as effect sizes according to $\eta^{2}$_p_ partial eta-squared.

Table 3: The effect of gender on the neuromuscular activity of the shank (A) and thigh (B) muscles from the different jump height low, middle and high for the respective phases short- (SLR), middle- (MLR) and late-latency response (LLR) and the particular muscles gastrocnemius lateralis (GL), tibialis anterior (TA), soleus (SOL), vastus medialis (VM), vastus lateralis (VL), rectus femoris (RF), biceps femoris (BF) and gluteus maximus (Gmax).

(A)

| **Shank Musculature** | | *Gender* | | *Low* | | | *Middle* | | *High* | | *Statistics – rmANOVA (P, F,*$\eta^{2}$_p_*)* | |
| --- | --- | --- | --- | --- | --- | --- | --- | --- | --- | --- | --- | --- |
|  |  |  | *Known* | | *Unknown* | *Cheat* | *Known* | *Unknown* | *Known* | *Unknown* | *Interaction effect: anticipation*gender* | *Interaction effect: drop height*gender* |
| **GL** | **PRE** | Male | 0.70 ± 0.374 | | 0.66 ± 0.366 | 0.65 ± 0.315 | 0.76 ± 0.410 | 0.72 ± 0.382 | 0.79 ± 0.396 | 0.68 ± 0.312 | F(1,20)=1.88, *p=*0.19, $\eta^{2}$_p_=0.31 | F(1.47,29.32)=1.49, *p=*0.24, $\eta^{2}$_p_=0.67 |
|  |  | Female | 0.91 ± 0.907 | | 0.88 ± 0.919 | 1.13 ± 0.993 | 0.96 ± 0.882 | 0.92 ± 0.904 | 0.99 ± 0.882 | 0.99 ± 0.847 |  |  |
|  | **SLR** | Male | 1.07 ± 0.588 | | 0.95 ± 0.511 | 0.87 ± 0.666 | 1.15 ± 0.572 | 1.04 ± 0.565 | 1.20 ± 0.588 | 1.17 ± 0.732 | F(1,20)=  1.31, *p=*0.27, $\eta^{2}$_p_=0.06 | F(1.08,21.67)=0.12, *p=*0.76, $\eta^{2}$_p_=0.01 |
|  |  | Female | 1.37 ± 0.799 | | 1.16 ± 0.839 | 1.16 ± 0.910 | 1.41 ± 1.02 | 1.27 ± 0.810 | 1.51 ± 1.23 | 1.44 ± 1.13 |  |  |
|  | **MLR** | Male | 1.08 ± 0.481 | | 0.86 ± 0.401 | 0.74 ± 0.467 | 1.24 ± 0.501 | 1.01 ± 0.510 | 1.28 ± 0.573 | 1.19 ± 0.710 | F(1,20)=  1.64, *p=*0.22, $\eta^{2}$_p_=0.08 | F(1.32,26.34)=0.39, *p=*0.6, $\eta^{2}$_p_=0.02 |
|  |  | Female | 1.32 ± 1.01 | | 1.01 ± 0.918 | 0.89 ± 0.901 | 1.43 ± 1.06 | 1.10 ± 0.855 | 1.42 ± 1.36 | 1.23 ± 1.083 |  |  |
|  | **LLR** | Male | 1.29 ± 0.746 | | 0.92 ± 0.391 | 0.71 ± 0.391 | 1.44 ± 0.615 | 1.12 ± 0.657 | 1.43 ± 0.635 | 1.34 ± 0.588 | F(1,20)=  0.05, *p=*0.83, $\eta^{2}$_p_=0.002 | F(1.5, 29.94)=0.7, *p=*0.46, $\eta^{2}$_p_=0.03 |
|  |  | Female | 1.58 ± 1.07 | | 1.25 ± 0.954 | 0.95 ± 0.725 | 1.86 ± 1.67 | 1.52 ± 1.22 | 1.67 ± 1.36 | 1.62 ± 1.41 |  |  |
| **TA** | **PRE** | Male | 0.42 ± 1.41 | | 0.39 ± 0.152 | 0.36 ± 0.153 | 0.45 ± 0.154 | 0.44 ± 0.152 | 0.48 ± 0.165 | 0.48 ± 0.165 | F(1,20)=  >0.001, *p=*0.99, $\eta^{2}$_p_<0.001 | F(1.36,27.26)= 1.41, *p=*0.26, $\eta^{2}$_p_=0.07 |
|  |  | Female | 0.47 ± 0.094 | | 0.44 ± 0.108 | 0.35 ± 0.175 | 0.50 ± 0.090 | 0.48 ± 0.092 | 0.53 ± 0.088 | 0.53 ± 0.095 |  |  |
|  | **SLR** | Male | 0.50 ± 0.191 | | 0.53 ± 0.198 | 0.52 ± 0.175 | 0.44 ± 0.223 | 0.50 ± 0.220 | 0.42 ± 0.229 | 0.42 ± 0.222 | F(1,20)=  0.95, *p=*0.34, η²_p_=0.05 | F(1.36,27.26)= 1.41, *p=*0.26, η²_p_=0.07 |
|  |  | Female | 0.52 ± 0.163 | | 0.56 ± 0.121 | 0.58 ± 0.203 | 0.49 ± 0.135 | 0.54 ± 0.171 | 0.48 ± 0.121 | 0.53 ± 0.138 |  |  |
|  | **MLR** | Male | 0.54 ± 0.271 | | 0.70 ± 0.301 | 0.98 ± 0.482 | 0.45 ± 0.236 | 0.54 ± 0.248 | 0.44 ± 0.245 | 0.44 ± 0.225 | F(1,20)=  0.07, *p=*0.8, $\eta^{2}$_p_=0.003 | F(1.22, 24.46)=  1.54, *p=*0.23, $\eta^{2}$_p_=0.07 |
|  |  | Female | 0.53 ± 0.197 | | 0.65 ± 0.221 | 0.70 ± 0.280 | 0.48 ± 0.210 | 0.57 ± 216 | 0.50 ± 0.205 | 0.51 ± 0.227 |  |  |
|  | **LLR** | Male | 0.45 ± 0.150 | | 0.58 ± 0.228 | 0.66 ± 0.305 | 0.40 ± 0.142 | 0.50 ± 0.227 | 0.41 ± 0.155 | 0.42 ± 0.173 | F(1,20)=  0.11, *p=*0.74, $\eta^{2}$_p_=0.006 | F(1.49, 29.78)=  0.5, *p=*0.56, $\eta^{2}$_p_=0.02 |
|  |  | Female | 0.49 ± 0.144 | | 0.59 ± 0.187 | 0.67 ± 0.181 | 0.46 ± 0.123 | 0.54 ± 0.157 | 0.48 ± 0.146 | 0.50 ± 0.169 |  |  |
| **SOL** | **PRE** | Male | 0.76 ± 0.536 | | 0.75 ± 0.541 | 0.76 ± 0.542 | 0.77 ± 0.524 | 0.76 ± 0.530 | 0.80 ± 0.511 | 0.78 ± 0.513 | F(1,20)=2.0, *p=*0.17, $\eta^{2}$_p_=0.09 | F(1.74,34.78)=  1.04, *p=*0.36, $\eta^{2}$_p_=0.05 |
|  |  | Female | 0.80 ± 0.393 | | 0.84 ± 0.473 | 0.86 ± 0.476 | 0.93 ± 0.520 | 0.83 ± 0.438 | 0.90 ± 0.450 | 0.80 ± 0.386 |  |  |
|  | **SLR** | Male | 1.15 ± 0.486 | | 0.99 ± 0.433 | 0.90 ± 0.475 | 1.29 ± 0.479 | 1.11 ± 0.456 | 1.33 ± 0.514 | 1.20 ± 0.497 | F(1,20)=  0.04, *p=*0.85, $\eta^{2}$_p_=0.002 | F(1.47, 29.4)=  1.63, *p=*0.22, $\eta^{2}$_p_=0.08 |
|  |  | Female | 1.46 ± 0.528 | | 1.20 ± 0.517 | 1.29 ± 0.824 | 1.47 ± 0.409 | 1.29 ± 0.526 | 1.44 ± 0.486 | 1.37 ± 0.427 |  |  |
|  | **MLR** | Male | 1.14 ± 0.553 | | 0.85 ± 0.576 | 0.79 ± 0.578 | 1.28 ± 0.464 | 0.98 ± 0.557 | 1.32 ± 0.501 | 1.26 ± 0.548 | F(1,20)=  0.38, *p=*0.54, $\eta^{2}$_p_=0.02 | F(1.5, 30.03)=  0.7, *p=*0.46, $\eta^{2}$_p_=0.03 |
|  |  | Female | 1.40 ± 0.359 | | 1.04 ± 0.317 | 0.92 ± 0.430 | 1.49 ± 0.490 | 1.16 ± 0.390 | 1.47 ± 0.430 | 1.36 ± 0.422 |  |  |
|  | **LLR** | Male | 1.34 ± 0.556 | | 1.04 ± 0.575 | 1.56 ± 0.540 | 1.56 ± 0.553 | 1.23 ± 0.582 | 1.57 ± 0.559 | 0.76 ± 0.599 | F(1,20)=  0.71, *p=*0.41, $\eta^{2}$_p_=0.03 | F(1.2, 24.0)=0.84, *p=*0.39, $\eta^{2}$_p_=0.04 |
|  |  | Female | 1.68 ± 0.439 | | 1.28 ± 0.368 | 0.95 ± 0.402 | 1.73 ± 0.325 | 1.60 ± 0.529 | 1.65 ± 0.457 | 1.69 ± 0.520 |  |  |

(B)

| **Thigh musculature** | | *Gender* | | | | *Low* | | | | *Middle* | | | *High* | | *Statistics – rmANOVA (P, F,*$\eta^{2}$_p_*)* | |
| --- | --- | --- | --- | --- | --- | --- | --- | --- | --- | --- | --- | --- | --- | --- | --- | --- |
|  |  |  | | *Known* | | *Unknown* | | *Cheat* | | *Known* | | *Unknown* | *Known* | *Unknown* | *Interaction effect: anticipation*gender* | *Interaction effect: drop height*gender* |
| **VM** | **PRE** | Male | 0.58 ± 0.337 | | 0.57 ± 0.336 | | 0.64 ± 0.418 | | 0.62 ± 0.339 | | 0.58 ± 0.329 | | 0.66 ± 0.365 | 0.58 ± 0.291 | F(1,20)=  0.28, *p=*0.6, $\eta^{2}$_p_=0.01 | F(1.23, 24.52)=1.16, *p=*0.31, $\eta^{2}$_p_=0.06 |
|  |  | Female | 0.55 ± 0.286 | | 0.53 ± 0.295 | | 0.63 ± 0.350 | | 0.60 ± 0.274 | | 0.56 ± 0.286 | | 0.64 ± 0.261 | 0.61 ± 0.251 |  |  |
|  | **SLR** | Male | 1.34 ± 0.632 | | 0.94 ± 0.373 | | 0.78 ± 0.360 | | 1.56 ± 0.649 | | 1.29 ± 0.535 | | 1.64 ± 0.816 | 1.56 ± 0.758 | **F(1,20)=4.58, *p=*0.05,** $\boldsymbol{\eta}^{\boldsymbol{2}}$**_p_=0.19** | **F(1.14,22.7)= 4.93, *p=*0.03,** $\boldsymbol{\eta}^{\boldsymbol{2}}$**_p_=0.2** |
|  |  | Female | 1.20 ± 0.323 | | 1.06 ± 0.311 | | 0.91 ± 0.411 | | 1.19 ± 0.311 | | 1.12 ± 0.339 | | 1.16 ± 0.380 | 1.16 ± 0.316 |  |  |
|  | **MLR** | Male | 1.62 ± 0.645 | | 1.24 ± 0.416 | | 1.04 ± 0.456 | | 1.77 ± 0.754 | | 1.63 ± 0.784 | | 1.68 ± 0.874 | 1.52 ± 0.781 | F(1,20)=4.33, *p=*0.05, $\eta^{2}$_p_=0.18 | F(1.76,35.27)=  0.62, *p=*0.52, $\eta^{2}$_p_=0.03 |
|  |  | Female | 1.31 ± 0.302 | | 1.20 ± 0.327 | | 1.29 ± 0.569 | | 1.42 ± 0.517 | | 1.33 ± 0.375 | | 1.35 ± 0.530 | 1.33 ± 0.477 |  |  |
|  | **LLR** | Male | 1.75 ± 0.778 | | 1.64 ± 0.613 | | 1.33 ± 0.638 | | 1.82 ± 0.830 | | 1.69 ± 0.769 | | 1.83 ± 0.960 | 1.83 ± 0.961 | F(1,20)=0.001, *p=*0.48, $\eta^{2}$_p_<0.001 | F(1.58,31.58)=  0.49, *p=*0.24, $\eta^{2}$_p_=0.07 |
|  |  | Female | 1.31 ± 0.626 | | 1.15 ± 0.358 | | 1.28 ± 0.348 | | 1.28 ± 0.788 | | 1.27 ± 0.349 | | 1.21 ± 0.649 | 1.15 ± 0.359 |  |  |
| **VL** | **PRE** | Male | 0.53 ± 0.273 | | 0.51 ± 0.267 | | 0.50 ± 0.247 | | 0.56 ± 0.274 | | 0.53 ± 0.261 | | 0.60 ± 0.280 | 0.57 ± 0.269 | F(1,20)=  3.58, *p=*0.07, $\eta^{2}$_p_=0.15 | F(1.12,22.33)=0.58, *p=*0.47, $\eta^{2}$_p_=0.03 |
|  |  | Female | 0.59 ± 0.370 | | 0.57 ± 0.382 | | 0.54 ± 0.382 | | 0.65 ± 0.359 | | 0.60 ± 0.368 | | 0.71 ± 0.381 | 0.59 ± 0.356 |  |  |
|  | **SLR** | Male | 1.16 ± 0.712 | | 0.85 ± 0.428 | | 0.83 ± 0.578 | | 1.46 ± 0.936 | | 0.99 ± 0.415 | | 1.33 ± 0.613 | 1.20 ± 0.567 | F(1,20)=  2.78, *p=*0.11, $\eta^{2}$_p_=0.12 | F(1.75,35.0)=  0.67, *p=*0.5, $\eta^{2}$_p_=0.03 |
|  |  | Female | 1.27 ± 0.611 | | 1.17 ± 0.657 | | 1.02 ± 0.652 | | 1.42 ± 0.815 | | 1.34 ± 0.814 | | 1.43 ± 0.822 | 1.30 ± 0.806 |  |  |
|  | **MLR** | Male | 1.11 ± 0.432 | | 0.95 ± 0.480 | | 0.81 ± 0.453 | | 1.26 ± 0.537 | | 1.13 ± 0.485 | | 1.18 ± 0.516 | 1.16 ± 0.442 | F(1,19)=4.31, *p=*0.05, $\eta^{2}$_p_=0.19 | F(1.41,26.73)=  0.05, *p=*0.9, $\eta^{2}$_p_=0.003 |
|  |  | Female | 1.40 ± 0.620 | | 1.34 ± 0.577 | | 1.21 ± 0.591 | | 1.47 ± 0.696 | | 1.55 ± 0.798 | | 1.51 ± 0.837 | 1.54 ± 0.959 |  |  |
|  | **LLR** | Male | 1.45 ± 0.804 | | 1.27 ± 0.663 | | 1.14 ± 0.711 | | 1.45 ± 0.847 | | 1.42 ± 0.842 | | 1.48 ± 1.01 | 1.45 ± 1.02 | F(1,20)=0.94, *p=*0.34, $\eta^{2}$_p_=0.05 | F(1.18,23.67)=  0.69, *p=*0.44, $\eta^{2}$_p_=0.03 |
|  |  | Female | 1.39 ± 0.963 | | 1.33 ± 0.827 | | 1.35 ± 0.951 | | 1.28 ± 0.863 | | 1.40 ± 0.772 | | 1.33 ± 0.635 | 1.31 ± 0.645 |  |  |
| **RF** | **PRE** | Male | 0.52 ± 0.333 | | 0.52 ± 0.325 | | 0.52 ± 0.325 | | 0.54 ± 0.336 | | 0.53 ± 0.330 | | 0.58 ± 0.351 | 0.56 ± 0.355 | F(1,20)=  2.06, *p=*0.17, $\eta^{2}$_p_=0.09 | F(1.08,21.66)=0.03, *p=*0.89, $\eta^{2}$_p_=0.001 |
|  |  | Female | 0.71 ± 0.703 | | 0.73 ± 0.700 | | 0.73 ± 0.704 | | 0.76 ± 0.701 | | 0.73 ± 0.702 | | 0.82 ± 0.717 | 0.73 ± 0.592 |  |  |
|  | **SLR** | Male | 1.11 ± 0.761 | | 0.78 ± 0.468 | | 0.72 ± 0.519 | | 1.33 ± 0.872 | | 1.08 ± 0.680 | | 1.32 ± 0.830 | 1.22 ± 0.680 | F(1,20)=  4.32, *p=*0.05, $\eta^{2}$_p_=0.18 | F(1.2,24.05)=  0.31, *p=*0.63, $\eta^{2}$_p_=0.02 |
|  |  | Female | 1.47 ± 0.807 | | 1.34 ± 0.889 | | 1.13 ± 1.282 | | 1.60 ± 1.043 | | 1.56 ± 0.966 | | 1.59 ± 1.08 | 1.68 ± 1.28 |  |  |
|  | **MLR** | Male | 2.06 ± 1.321 | | 1.92 ± 1.08 | | 1.76 ± 1.18 | | 1.96 ± 1.39 | | 1.97 ± 1.19 | | 1.99 ± 1.75 | 2.05 ± 1.82 | F(1,20)<0.001, *p=*0.98, $\eta^{2}$_p_<0.001 | F(1.48,29.61)=  0.3, *p=*0.68, $\eta^{2}$_p_=0.02 |
|  |  | Female | 2.23 ± 1.76 | | 2.14 ± 1.39 | | 2.40 ± 1.77 | | 2.26 ± 2.05 | | 2.30 ± 1.86 | | 2.18 ± 1.51 | 2.16 ± 1.31 |  |  |
|  | **LLR** | Male | 1.47 ± 0.944 | | 1.37 ± 0.770 | | 1.26 ± 0.840 | | 1.40 ± 0.993 | | 1.40 ± 0.852 | | 1.42 ± 1.25 | 1.46 ± 1.297 | F(1,20)=0.001, *p=*0.98, $\eta^{2}$_p_<0.001 | F(1.48,29.59)=  0.29, *p=*0.68*,* $\eta^{2}$_p_=0.02 |
|  |  | Female | 1.59 ± 1.26 | | 1.53 ± 0.992 | | 1.71 ± 1.26 | | 1.61 ± 1.46 | | 1.64 ± 1.33 | | 1.56 ± 1.08 | 1.54 ± 0.933 |  |  |
| **BF** | **PRE** | Male | 0.28 ± 0.134 | | 0.25 ± 0.127 | | 0.24 ± 0.117 | | 0.29 ± 0.153 | | 0.28 ± 0.147 | | 0.30 ± 0.171 | 0.29 ± 0.172 | F(1,20)=2.9, *p=*0.1, $\eta^{2}$_p_=0.13 | F(1.13,22.62)=0.26, *p=*0.65*,* $\eta^{2}$_p_=0.01 |
|  |  | Female | 0.39 ± 0.225 | | 0.37 ± 0.232 | | 0.35 ± 0.199 | | 0.40 ± 0.234 | | 0.39 ± 0.226 | | 0.39 ± 0.223 | 0.40 ± 0.241 |  |  |
|  | **SLR** | Male | 0.34 ± 0.129 | | 0.32 ± 0.169 | | 0.40 ± 0.285 | | 0.35 ± 0.122 | | 0.31 ± 0.107 | | 0.40 ± 0.160 | 0.38 ± 0.154 | F(1,20)=  0.74, *p=*0.4, $\eta^{2}$_p_=0.04 | F(1.4,28.01)=0.12, *p=*0.81*,* $\eta^{2}$_p_=0.01 |
|  |  | Female | 0.44 ± 0.221 | | 0.39 ± 0.215 | | 0.37 ± 0.192 | | 0.47 ± 0.269 | | 0.38 ± 0.154 | | 0.49 ± 0.243 | 0.49 ± 0.253 |  |  |
|  | **MLR** | Male | 0.36 ± 0.205 | | 0.35 ± 0.193 | | 0.32 ± 0.240 | | 0.36 ± 0.169 | | 0.36 ± 0.184 | | 0.41 ± 0.160 | 0.41 ± 0.212 | F(1,20)=1.2, *p=*0.29, $\eta^{2}$_p_=0.06 | F(1.32,26.31)=0.14, *p=*0.78*,* $\eta^{2}$_p_=0.01 |
|  |  | Female | 0.40 ± 0.170 | | 0.39 ± 0.193 | | 0.46 ± 0.256 | | 0.45 ± 0.164 | | 0.38 ± 0.192 | | 0.49 ± 0.187 | 0.47 ± 0.205 |  |  |
|  | **LLR** | Male | 0.34 ± 0.146 | | 0.32 ± 0.186 | | 0.26 ± 0.151 | | 0.43 ± 0.199 | | 0.38 ± 0.194 | | 0.45 ± 0.196 | 0.47 ± 0.251 | F(1,20)=1.41, *p=*0.25, $\eta^{2}$_p_=0.07 | F(1.62,32.46)=1.2, *p=*0.31*,* $\eta^{2}$_p_=0.06 |
|  |  | Female | 0.49 ± 0.256 | | 0.41 ± 0.266 | | 0.32 ± 0.152 | | 0.53 ± 0.255 | | 0.47 ± 0.239 | | 0.54 ± 0.308 | 0.52 ± 0.311 |  |  |
| **Gmax** | **PRE** | Male | 0.54 ± 0.344 | | 0.54 ± 0.365 | | 0.54 ± 0.352 | | 0.60 ± 0.438 | | 0.56 ± 0.369 | | 0.58 ± 0.388 | 0.57 ± 0.371 | F(1,20)=0.37, *p=*0.55, $\eta^{2}$_p_=0.02 | F(1.14,22.83)=0.51, *p*=0.5*,* $\eta^{2}$_p_=0.03 |
|  |  | Female | 0.42 ± 0.331 | | 0.41 ± 0.338 | | 0.41 ± 0.344 | | 0.44 ± 0.323 | | 0.42 ± 0.334 | | 0.44 ± 0.318 | 0.45 ± 0.317 |  |  |
|  | **SLR** | Male | 0.75 ± 0.339 | | 0.66 ± 0.513 | | 0.61 ± 0.264 | | 0.91 ± 0.775 | | 0.73 ± 0.498 | | 0.77 ± 0.276 | 0.90 ± 0.844 | F(1,20)=0.49, *p=*0.49, $\eta^{2}$_p_=0.02 | F(1.83,36.55)=0.05,  *p=*0.95, $\eta^{2}$_p_=0.002 |
|  |  | Female | 0.78 ± 0.408 | | 0.62 ± 0.322 | | 0.54 ± 0.415 | | 0.84 ± 0.410 | | 0.76 ± 0.418 | | 0.85 ± 0.424 | 0.81 ± 0.386 |  |  |
|  | **MLR** | Male | 0.99 ± 0.656 | | 0.85 ± 0.704 | | 0.74 ± 0.582 | | 1.12 ± 0.813 | | 1.01 ± 0.897 | | 1.04 ± 0.507 | 1.05 ± 0.843 | F(1,20)=  0.64, *p=*0.43, $\eta^{2}$_p_=0.03 | F(1.86,37.86)=  0.84, *p=*0.43, $\eta^{2}$_p_=0.04 |
|  |  | Female | 0.97 ± 0.519 | | 0.82 ± 0.407 | | 0.74 ± 0.438 | | 1.03 ± 0.506 | | 0.85 ± 0.463 | | 0.99 ± 0.515 | 0.93 ± 0.466 |  |  |
|  | **LLR** | Male | 0.84 ± 0.437 | | 0.76 ± 0.316 | | 0.81 ± 0.4580.85 | | 0.92 ± 0.469 | | 0.79 ± 0.258 | | 0.91 ± 0.465 | 0.90 ± 0.206 | F(1,20)=1.18, *p=*0.29, $\eta^{2}$_p_=0.06 | F(1.75,34.96)= 0.82,  *p=*0.44, $\eta^{2}$_p_=0.04 |
|  |  | Female | 0.85 ± 0.422 | | 0.79 ± 0.368 | | 0.60 ± 0.316 | | 0.88 ± 0.405 | | 0.93 ± 0.451 | | 0.93 ± 0.400 | 1.03 ± 0.492 |  |  |

Bold values indicate significant interaction effects of the rmANOVA (*p* < 0.05). Values are means ± SD. The interaction effects between anticipation * gender and drop height * gender are displayed with corresponding *F* values and *p* values (**p* < 0.05), as well as effect sizes according to $\eta^{2}$_p_ partial eta-squared.
